# Outbreak of porcine deltacoronavirus in pigs in central China, 2024

**DOI:** 10.3389/fvets.2026.1934473

**Published:** 2026-09-30

**Authors:** Guoliang Chen, Haichao Wu, Lingfeng Li, Bo Wang, Juanjuan Min, Yonghuan Deng, Can Yin, Ping Zhou, Yushuang Zhou, Wen Sun, Taotao Yang, Bo Ma, Yuli Hu

**Affiliations:** 1 Huaihua Vocational and Technical College, Huaihua, Hunan, China; 2 Sinopharm Animal Health Corporation Ltd., Wuhan, Hubei, China; 3 School of Life Sciences and Environmental Resources, Yichun University, Yichun, Jiangxi, China; 4 Wuhan Academy of Agricultural Sciences, Wuhan, Hubei, China

**Keywords:** amino acid substitution, China, outbreak, porcine deltacoronavirus, spike protein

## Abstract

Porcine deltacoronavirus (PDCoV) is an emerging enteropathogenic coronavirus responsible for acute diarrhea, vomiting, and dehydration in piglets, thereby posing a substantial threat to the global swine industry. In December 2024, a severe diarrheal disease outbreak occurred on an intensive pig farm in Hubei Province, located in central China. Here, we describe the isolation and genomic characterization of a novel PDCoV strain, designated SP2403-2, obtained from the intestinal contents of an affected piglet. The virus was successfully propagated in cell culture and confirmed by immunofluorescence staining and transmission electron microscopy. The near-complete genome of SP2403-2, comprising 25,244 nucleotides, was sequenced. Comparative genomic analyses showed that SP2403-2 shares 97.0–99.2% nucleotide identity with reference PDCoV strains, with the highest similarity (99.2%) to the recently reported Chinese strain JSTZ2025. Phylogenetic analyses based on complete genome, ORF1ab, and S gene sequences demonstrated that SP2403-2 belongs to the Chinese lineage. Notably, alignment of the spike (S) protein of SP2403-2 with that of the vaccine strain CHN-HN-2014 revealed 25 amino acid substitutions. Structural mapping indicated that these mutations are predominantly located on the surface-exposed regions of critical domains within the S protein, potentially influencing antigenic epitopes and receptor-binding properties. Our findings demonstrate that SP2403-2 represents a contemporary Chinese-lineage PDCoV strain carrying substantial S protein variation relative to the vaccine strain, underscoring the imperative for ongoing surveillance of emerging PDCoV variants and re-evaluation of current vaccine efficacy.

## Introduction

Porcine deltacoronavirus (PDCoV) is a novel enteropathogenic coronavirus classified within the genus *Deltacoronavirus* of the family *Coronaviridae* (1). Initially identified in Hong Kong in 2012 during a regional surveillance effort (2), PDCoV has since become a significant pathogen affecting swine herds globally (3). The virus induces acute watery diarrhea, vomiting, dehydration, and mortality in piglets, particularly in neonates, leading to considerable economic losses for the pork industry worldwide (4).

Following its initial detection, PDCoV has been documented in multiple countries across North America, Asia, and South America, including the United States, Canada, South Korea, China, Thailand, Vietnam, Laos, Japan, Peru, and Mexico (5–13). In China, PDCoV was first identified on the mainland in 2015 from diarrheal samples collected in Jiangxi Province (14). Subsequent epidemiological surveys have demonstrated that PDCoV is widely prevalent among Chinese pig populations (15–19). Moreover, PDCoV infections are frequently detected as coinfections with other enteric pathogens, particularly porcine epidemic diarrhea virus (PEDV), which may exacerbate clinical severity (16, 20).

The PDCoV genome is a positive-sense, single-stranded RNA of approximately 25.4 kb, encoding four structural proteins—spike (S), envelope (E), membrane (M), and nucleocapsid (N)—alongside several nonstructural proteins (nsps) and accessory proteins (NS6, NS7, and NS7a) (1). Among these, the S protein plays a pivotal role in viral entry by mediating receptor binding and membrane fusion, and it serves as the primary target for host neutralizing antibodies (21–23). The S protein is divided into two subunits: S1, which contains the receptor-binding domains (S1-NTD and S1-CTD), and S2, which facilitates membrane fusion (24). The S1-NTD and S1-CTD have been identified as regions harboring major neutralizing epitopes (25, 26).

Despite advances in diagnostic tools and an expanding understanding of PDCoV epidemiology, effective vaccines and specific antiviral therapies remain limited (27). In China, the PDCoV vaccine strain CHN-HN-2014 has been employed for immunization; however, the antigenic variability of field strains may compromise vaccine efficacy (28). Consequently, continuous surveillance of emerging PDCoV strains is essential to monitor viral evolution and evaluate potential immune escape.

In December 2024, a severe diarrheal outbreak occurred in an intensive pig farm in Hubei Province, central China, characterized by high morbidity and mortality among neonatal piglets. In this report, we present the isolation, genomic characterization, and phylogenetic analysis of a novel PDCoV strain, SP2403-2, derived from this outbreak. Our findings provide important insights into the genetic diversity and antigenic variation of contemporary PDCoV strains circulating in China.

## Materials and methods

### Clinical outbreak and pathogen detection

In December 2024, a severe diarrheal outbreak took place on an intensive pig farm in Hubei Province, central China, which housed approximately 2,100 sows. The farm had implemented a routine vaccination program against porcine epidemic diarrhea virus (PEDV) and transmissible gastroenteritis virus (TGEV) using commercial inactivated vaccines, administered to sows twice during gestation. However, no specific PDCoV vaccine had been employed on this farm prior to the outbreak. The outbreak initiated with sows in one farrowing unit (80 sows) displaying diarrheal signs. Piglets began showing clinical symptoms at 3 days of age, including acute watery diarrhea, dehydration, and lethargy. The mortality rate among affected piglets was approximately 20%, corresponding to an estimated 240 piglet deaths and economic losses of at least 140,000 RMB.

Intestinal tissue samples were collected from a diseased piglet exhibiting acute watery diarrhea. Nucleic acids were extracted using an automated extraction system (TIANLONG, China). Samples were tested by reverse transcription-quantitative PCR (RT-qPCR) and qPCR (Takara, China) for the presence of PEDV, porcine deltacoronavirus (PDCoV), TGEV, porcine rotavirus (PoRV), Getah virus (GETV), and porcine circovirus type 2 (PCV2) using specific primer and probe sets.

### Virus isolation and propagation

Intestinal homogenates from the PDCoV-positive piglet were clarified by centrifugation (12,000 rpm, 20 min) and filtered through a 0.22 μm filter. The filtrate was inoculated onto confluent monolayers of LLC-PK1 cells (CCTCC, China, No. GDC0612) maintained in Dulbecco’s Modified Eagle Medium (DMEM) supplemented with 2.5 μg/mL trypsin (Solarbio, China). After adsorption at 37 °C for 1 h, the inoculum was removed, and cells were washed with phosphate-buffered saline and maintained in DMEM containing 2.5 μg/mL trypsin. Cultures were monitored daily for cytopathic effects (CPE). Following three freeze–thaw cycles and centrifugation (4,000 rpm, 5 min), the supernatant was passaged onto fresh LLC-PK1 cells for seven consecutive passages. The isolated virus strain was stored at −80 °C for subsequent analysis.

### Replication kinetics analysis

To evaluate the growth characteristics of the isolated virus, LLC-PK1 monolayers in 6-well plates were infected at a multiplicity of infection (MOI) of 0.01. After adsorption at 37 °C for 1 h, cells were washed with PBS and maintained in DMEM containing 2.5 μg/mL trypsin. Culture supernatants were collected at 12, 24, 36, 48, 60, 72, 84, and 96 h post-infection (hpi) in triplicate, and viral titers were determined by the 50% tissue culture infectious dose (TCID_50_) assay using the Reed-Muench method.

### Immunofluorescence assay

LLC-PK1 cells infected with the isolated virus at an MOI of 0.01 were fixed with 4% glutaraldehyde at 24 h post-infection (hpi) for 15 min, followed by permeabilization with 0.2% Triton X-100 for 10 min. Cells were incubated with PDCoV-specific polyclonal antibodies (Gifted by China agricultural university which prepared in-house by immunizing rabbits with purified inactivated PDCoV CHN-HN-1601 strain particles), followed by FITC-conjugated goat anti-mouse IgG (1,800, Thermo Scientific, USA). Fluorescence signals were visualized using a fluorescence microscope (Zeiss Axio Observer, Germany).

### Transmission electron microscopy

Culture supernatants from PDCoV-infected LLC-PK1 cells were concentrated by centrifugation (4,000 rpm, 5 min) and filtered through a 0.22 μm filter. The filtrate was negatively stained with 2% phosphotungstic acid and examined using a transmission electron microscope (Hitachi HC-1, Japan).

### Genome sequencing and assembly

Viral RNA was extracted from the culture supernatant and reverse-transcribed using the RevertAid First Strand cDNA Synthesis Kit (Thermo Scientific, USA). The complete genome of PDCoV was amplified using Pfu DNA polymerase (Vazyme, China) with previously reported primer pairs (29). Sequencing was performed using the Sanger method. To minimize sequencing errors inherent to Sanger sequencing, the complete genome was amplified using high-fidelity Pfu DNA polymerase, which possesses 3′ → 5′ exonuclease proofreading activity. Each amplified fragment was sequenced in both directions using overlapping primers. Sequence assembly was performed with independent sequencing reads covering each nucleotide position to ensure consensus accuracy. Ambiguous bases were manually curated by referring to the raw chromatogram data. Sequence assembly and multiple alignments were carried out using Lasergene software (DNASTAR, Madison, WI, USA) (30).

### Phylogenetic analysis

Phylogenetic trees were constructed based on the complete genome, ORF1ab, and S gene sequences using MEGA 6.0.6 software (31). The maximum-likelihood (ML) method was employed with the General Time Reversible (GTR) substitution model incorporating gamma-distributed rate heterogeneity and a proportion of invariant sites (G + I) in this study. Bootstrap support was assessed with 1,000 replicates, and values greater than 70% are shown on the phylogenetic trees. The ML method was preferred over the neighbor-joining (NJ) method for phylogenetic tree construction because ML employs a statistical framework that incorporates a more complex and realistic model of nucleotide substitution (GTR + G + I). This approach is generally considered more robust for inferring evolutionary relationships, especially when analyzing sequences with varying rates of evolution, as is common in RNA viruses like PDCoV.

### Structural analysis of the spike protein

The cryo-electron microscopy structure of the PDCoV S protein (PDB file: 6B7N) was visualized using PyMOL software (Molecular Graphics System, version 2.4, Schrödinger, LLC) (24). Amino acid substitutions identified in the isolated strain relative to the vaccine strain CHN-HN-2014 were mapped onto the three-dimensional structure of the S protein trimer.

## Results

### Pathogen detection

RT-qPCR and qPCR testing of intestinal samples from the diseased piglet confirmed the presence of PDCoV as the sole detectable enteric pathogen, whereas PEDV, TGEV, PoRV, GETV, and PCV2 were all negative (Supplementary Figure S1). These results indicated that PDCoV was the primary etiological agent responsible for this outbreak.

### Virus isolation and characterization

The PDCoV-positive intestinal homogenate was successfully propagated in LLC-PK1 cells. After three serial passages, characteristic CPE were observed, initially appearing as cell rounding and shrinkage at 24 hpi, progressing to cell detachment and complete CPE formation at 48–72 hpi (Figure 1A). The isolated virus was designated SP2403-2 and was serially propagated for seven passages in LLC-PK1 cells.

**Figure 1:**
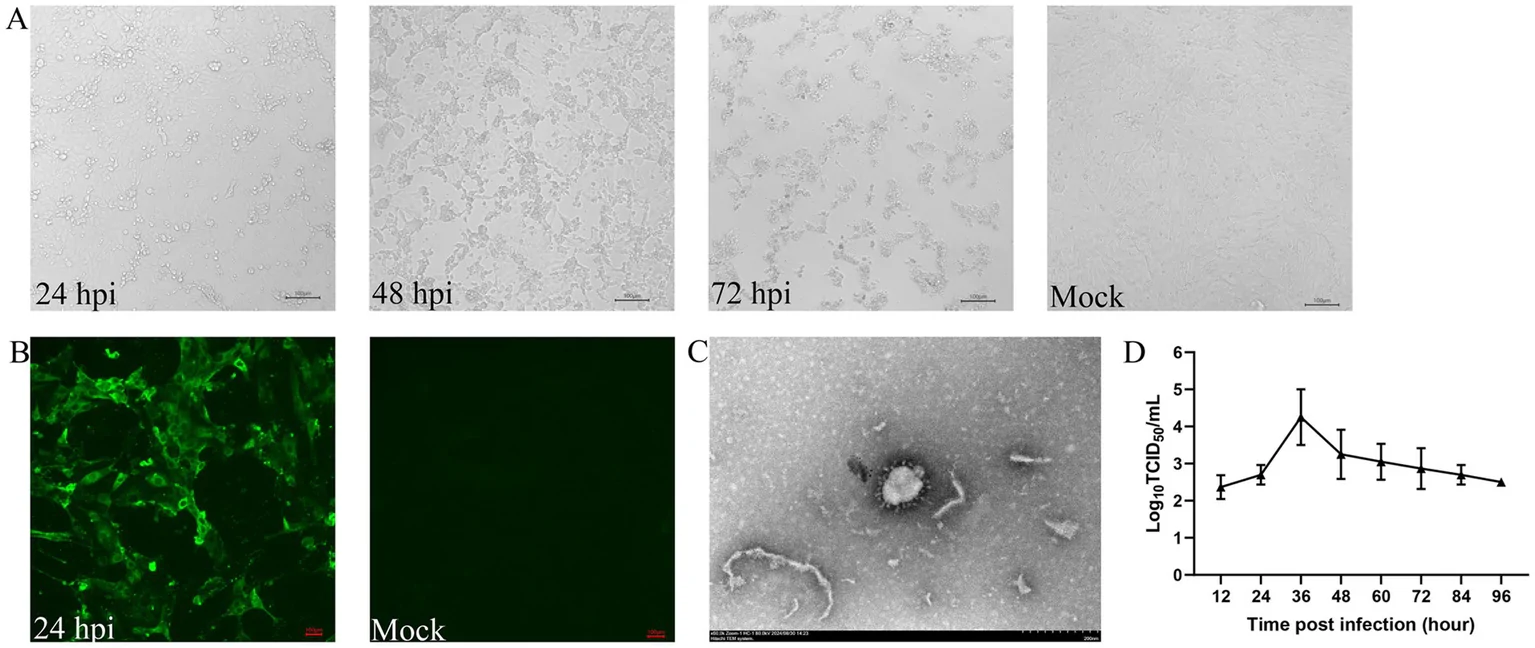
Isolation and characterization of PDCoV strain SP2403-2. **(A)** Cytopathic effects (CPE) observed in LLC-PK1 cells at 24, 48, and 72 h post-infection (hpi) with PDCoV SP2403-2, compared to mock-infected control cells. Scale bars represent 100 μm. **(B)** Immunofluorescence detection of PDCoV in SP2403-2-infected LLC-PK1 cells at 24 hpi and negative control (mock-infected cells). Scale bars represent 100 μm. **(C)** Transmission electron micrograph of PDCoV SP2403-2 particles showing characteristic crown-shaped spikes (inset). Samples were negatively stained with 2% phosphotungstic acid. Scale bar: 200 nm. **(D)** Replication kinetics of PDCoV SP2403-2 in LLC-PK1 cells. Viral titers (TCID_50_/mL) were measured at the indicated time points. Data are presented as mean ± standard deviation (SD) from three independent replicates.

Immunofluorescence assay using PDCoV-specific polyclonal antibodies confirmed viral replication in infected LLC-PK1 cells at 24 hpi, with distinct green fluorescence signals detected in the cytoplasm of infected cells, whereas no fluorescence was observed in mock-infected control cells (Figure 1B).

Transmission electron microscopy of negatively stained culture supernatants revealed typical coronavirus particles (Figure 1C), which were spherical with diameters of 100–120 nm and possessed characteristic surface projections (spikes), consistent with the morphology of PDCoV.

To characterize the replication kinetics of SP2403-2, viral titers were determined at various time points post-infection. The virus titer increased from 12 hpi, reached a peak of 10^4.25^ TCID_50_/mL at 36 hpi, and followed by a gradual decline (Figure 1D). This replication kinetics profile indicating that SP2403-2 exhibits replicative fitness in LLC-PK1 cells.

### Genomic characteristics of SP2403-2

The near-complete genome of PDCoV SP2403-2 was successfully sequenced, comprising 25,244 nucleotides (GenBank accession no. PZ671938). The genome organization included all expected coding regions: ORF1ab (18,799 bp, encoding 4,209 amino acids), S (3,480 bp, 1,159 amino acids), E (252 bp, 83 amino acids), M (654 bp, 217 amino acids), NS6 (285 bp, 94 amino acids), N (1,029 bp, 342 amino acids), and NS7 (603 bp, 200 amino acids).

Comparative genomic analysis revealed that the complete genome of SP2403-2 shares 97.0–99.2% nucleotide identity with reference PDCoV strains (Table 1). Nucleotide BLAST analysis showed the highest similarity (99.2%) to the recent Chinese strain JSTZ2025 (GenBank: PX126649). Detailed sequence comparisons for each gene are summarized in Table 1.

**Table 1:** Homology comparison between the PDCoV SP2403-2 isolate and PDCoV reference isolates previously reported in different country.

Strains	% nucleotide and deduced amino acid identities														
	Complete genome	ORF 1a/b		S		E		M		NS6		N		NS7	
	nt^a^	nt	aa^b^	nt	aa	nt	aa	nt	aa	nt	aa	nt	aa	nt	aa
China	98.0–99.2	98.2–99.1	98.9–99.8	96.7–98.5	96.5–99.3	98.4–100	98.8–100	98.6–99.8	97.7–100	97.9–100	95.7–100	97.9–99.5	98.8–99.7	98.2–99.8	95.0–99.5
USA	98.2–98.3	98.4–98.5	99.1–99.2	97.2–97.3	97.4–97.9	99.2–100	100	98.2–98.6	98.6–99.1	98.6–99.3	96.8–98.9	98.3–98.6	99.4–99.7	98.8–99.2	97.0–98.0
South Korea	97.8–98.2	98.1–98.4	99.0–99.2	96.5–97.1	96.7–97.5	99.2–99.6	98.8–100	98.5–98.6	99.1	98.6–98.9	97.9–98.9	98.0–98.4	99.4–99.7	98.3–98.8	95.5–96.5
Haiti	98.1	98.2	99.2	97.4	97.9	99.2	100	98.6	99.1	99.3	97.9	98.5	99.7	99.0	97.0
Vietnam	97.6	97.7–97.8	98.8	96.2	97.3–97.4	99.6	100	98.9	99.1	98.9	98.9	98.4	99.7	98.8	96.5
Thailand	97.0–97.7	97.3–97.8	97.9–98.9	95.2–97.0	96.2–97.4	99.6–100	100	98.3–98.5	98.6–99.5	97.9–98.2	97.9	97.4–97.8	98.2–98.8	97.2–97.7	92.5–94.5
Laos	97.3	97.6	98.6	95.7	96.7	100	100	98.3	98.6	98.2	98.9	97.6	98.5	97.5	93.5

a Nucleotide sequence identities.

b Deduced amino acid sequence identities.

### Phylogenetic analysis

Phylogenetic trees were constructed based on the complete genome, ORF1ab, and S gene sequences (Figure 2). All three phylogenetic analyses consistently demonstrated that SP2403-2 clusters closely with the Chinese strain JSTZ2025, confirming its classification within the Chinese lineage. Notably, SP2403-2 was genetically distinct from strains originating from the United States, South Korea, Haiti, and Southeast Asian countries (Vietnam, Thailand, and Laos), indicating that PDCoV strains exhibit significant geographical clustering with limited intercontinental transmission.

**Figure 2:**
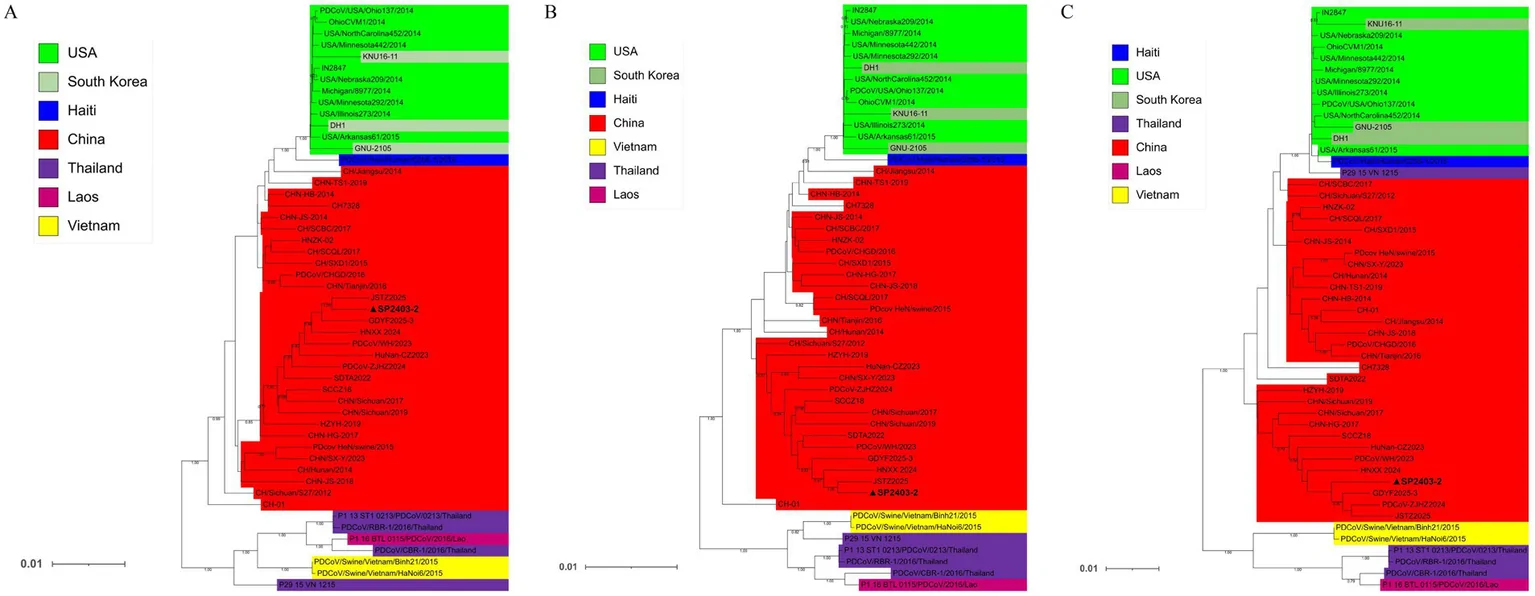
Evolutionary relationships of PDCoV based on gene sequences. **(A)** Complete genome, **(B)** ORF1ab, and **(C)** S gene. The strain identified in the present study (SP2403-2) is indicated by a triangle. The evolutionary history was inferred using the maximum-likelihood method with the General Time Reversible (GTR) model with gamma-distributed rates and proportion of invariant sites (G + I) using MEGA 6.06. The percentage of replicates in which the associated virus clustered together in the bootstrap test (1,000 replicates) is shown next to the branches (only values > 70% are shown) in each tree. The scale bar indicates nucleotide substitutions per site. PDCoV reference strains used in the study are shown in Supplementary Table S1.

### Amino acid substitutions in the spike protein and structural mapping

To characterize the molecular features of the SP2403-2 S protein, we compared its amino acid sequence with that of the Chinese vaccine strain CHN-HN-2014 (GenBank accession no. KT336560). A total of 25 amino acid substitutions were identified: P38L, R40S, S43T, S44T, Y46H, L93F, Q106L, D109E, I110V, H122Y, T135I, H148R, F171I, H228Q, A234V, V325I, K533E, Q641R, L665I, L669I, K800Q, T999N, D1085E, R1089K, and I1100V (Figure 3B).

**Figure 3:**
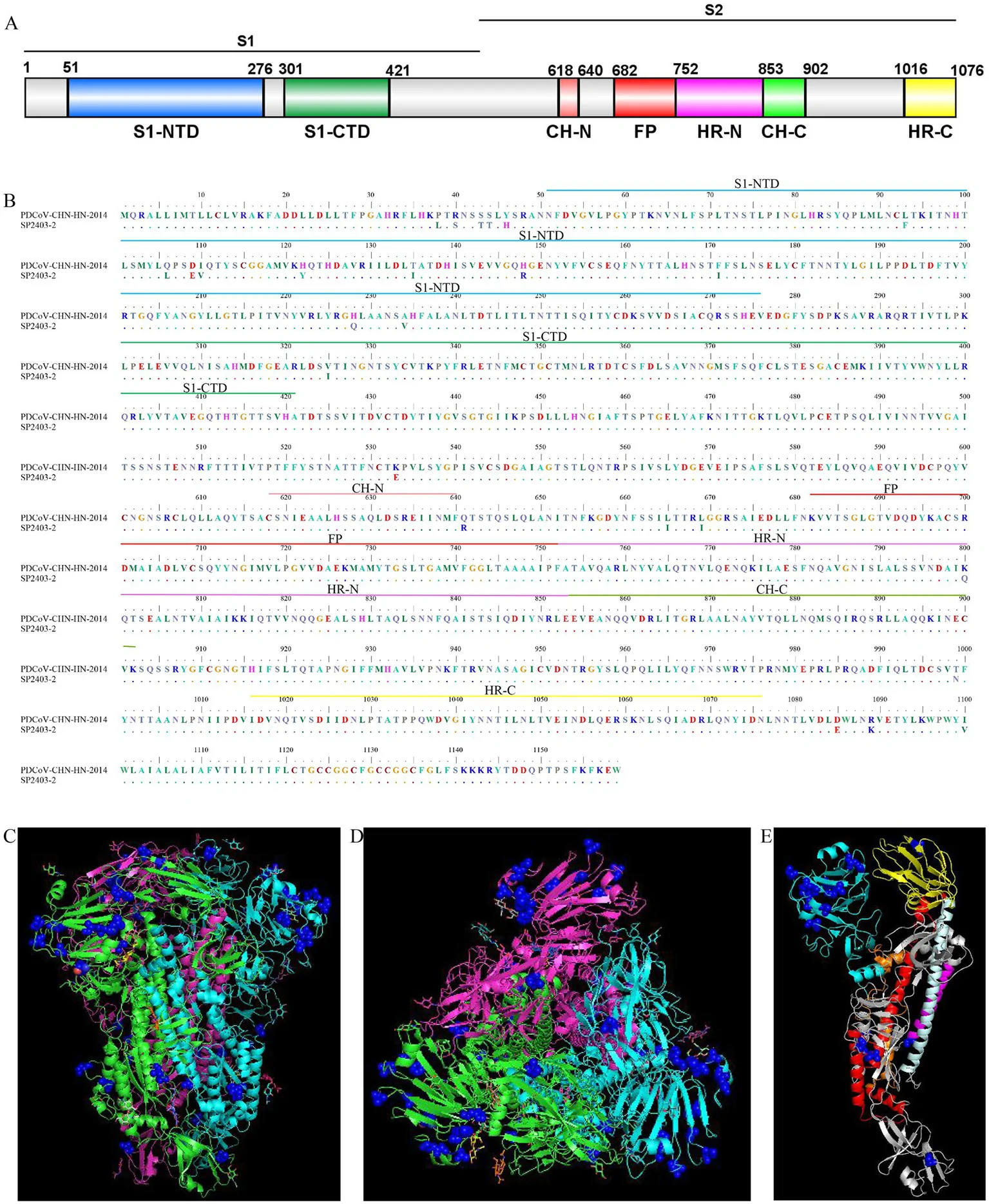
Amino acid substitutions and structural modeling in the spike protein of SP2403-2. **(A)** Schematic diagram of the PDCoV spike (S) protein. S1, receptor-binding subunit; S2, membrane fusion subunit; S1-NTD, N-terminal domain of S1; S1-CTD, C-terminal domain of S1; CH-N and CH-C, central helices N and C; FP, fusion peptide; HR-N and HR-C, heptad repeats N and C. **(B)** Comparative analysis of amino acid variations in S protein between PDCoV strain SP2403-2 and vaccine strain CHN-HN-2014 (GenBank accession no. KT336560). Multiple sequence alignment was performed using MEGA 6.06, and the figure was generated with BioEdit software (v7.2.5). **(C–E)** Structural mapping of amino acid variations between SP2403-2 and vaccine strain CHN-HN-2014 in the PDCoV S protein. **(C)** Side view and **(D)** top view of the S protein trimer, with chain A shown in green, chain B in magenta, and chain C in cyan. Mutated residues are depicted as blue spheres. **(E)** S protein monomer structure showing subdomains: S1-NTD (cyan), S1-CTD (yellow), CH-H (magenta), FP (orange), HR-N (red), and CH-C (pale cyan). Mutated residues are depicted as blue spheres.

These residues were mapped onto the cryo-EM structure of the PDCoV S protein trimer (Figures 3C,D). The S protein subunit comprises seven structural domains: S1-NTD, S1-CTD, CH-N, FP, HR-N, CH-C, and HR-C (Figures 3A,E). Notably, residues at positions 93, 106, 109, 110, 122, 135, 148, 171, 228, and 234 are located within the S1-NTD region and are exposed on the surface of the trimeric spike (Figure 3E). The residue at position 325 is situated at the apex of the S1-CTD and is also surface-exposed. In contrast, residue 800 is embedded within the HR-N region between two α-helices, while other substitutions are located in non-domain regions (Figure 3E). These structural data suggest that the observed amino acid substitutions may influence viral biological properties by altering surface epitopes or protein–protein interactions.

## Discussion

PDCoV has been widely distributed across China since its first report on the mainland in 2015 (15–19). A systematic review and meta-analysis estimated the overall prevalence of PDCoV infection in Chinese pigs at 12.2%, with central China showing a notably higher prevalence of 24.5% (32). During the period from 2014 to 2021, the highest prevalence was observed in 2015 at 20.5%, declining to 4.8% in 2021 (32). The outbreak reported in this study, occurring as recently as December 2024, underscores the continued and persistent threat posed by PDCoV to the Chinese swine industry, particularly in the high-prevalence central region. This is one of the first documented outbreak strains from 2024, providing a crucial snapshot of the contemporary circulating viral population. The isolation and characterization of SP2403-2 are significant because they offer an up-to-date reference for evaluating the genetic and antigenic drift of PDCoV in China, directly informing the need for updated diagnostic assays and vaccine strategies.

Phylogenetic analysis based on complete genome, ORF1ab, and S gene sequences consistently demonstrated that SP2403-2 belongs to the Chinese lineage and clusters closely with the recent Chinese strain JSTZ2025. PDCoV strains from different countries are typically categorized into distinct lineages based on S gene sequences, including China, Early China, the United States, and Southeast Asia lineages (33). The geographical clustering observed in our phylogenetic analysis—with SP2403-2 being genetically distinct from strains originating from the United States, South Korea, Haiti, and Southeast Asian countries—is consistent with the notion that PDCoV exhibits regional epidemiological characteristics. However, the close evolutionary relationship between certain geographically distant strains, such as those from the United States and South Korea, suggests that international trade and pig movements may facilitate transboundary virus transmission. These findings highlight the importance of strict biosecurity measures and surveillance at national borders.

The S protein of PDCoV plays a critical role in viral entry by mediating receptor binding and membrane fusion, and serves as the primary target for neutralizing antibodies (21–23). Previous studies have identified that the S1-CTD region may contain major neutralizing epitopes, while the S1-NTD has also been implicated in neutralizing antibody induction (25, 26). In this study, comparison of the SP2403-2 S protein with that of the Chinese vaccine strain CHN-HN-2014 revealed 25 amino acid substitutions, with most located within the S1-NTD and exposed on the surface of the trimeric spike structure, suggesting they may directly influence antibody binding or receptor interactions. The S1-NTD has been shown to be a critical domain for the induction of neutralizing antibodies (34, 35). The accumulation of mutations in neutralizing epitopes may indicate antigenic drift, potentially leading to immune escape and reduced vaccine efficacy. Given that the vaccine strain CHN-HN-2014 has been used for immunization in China, the substantial S protein variation observed in SP2403-2 raises important questions regarding the protective efficacy of current vaccines against emerging field strains. Continuous monitoring of antigenic variation in circulating PDCoV strains is therefore essential for assessing the need for vaccine updates.

The findings of this study have several implications for PDCoV control. First, the continued circulation of PDCoV in Chinese pig farms, as exemplified by this outbreak, underscores the need for enhanced biosecurity measures and surveillance programs. Second, the substantial S protein variation observed in SP2403-2 compared to the vaccine strain highlights the importance of evaluating the cross-protective efficacy of current vaccines against emerging field strains. Third, the surface-exposed nature of many identified substitutions suggests that these mutations may have functional significance in immune evasion, warranting further investigation through neutralization assays.

## Conclusion

In this study, we report the isolation and genomic characterization of a novel PDCoV strain, SP2403-2, from a severe diarrheal outbreak in Hubei Province, central China, in December 2024. Phylogenetic analysis confirmed that SP2403-2 belongs to the Chinese lineage. Notably, comparison with the vaccine strain CHN-HN-2014 revealed 25 amino acid substitutions in the S protein, with most located in the surface-exposed S1-NTD region, which is critical for neutralizing antibody induction. These findings suggest that contemporary PDCoV field strains are undergoing antigenic evolution, potentially affecting vaccine efficacy. The unique combination of mutations identified in this study, especially those located in the S1-NTD, defines a distinct genetic signature for SP2403-2 that can be used as a molecular marker for surveillance. The updated epidemiological and genetic data provided here are vital for risk assessment and for designing next-generation vaccines with broader cross-protection against emerging variants. Continuous surveillance of PDCoV genetic diversity and evaluation of cross-neutralization between vaccine strains and emerging field isolates are urgently needed to inform vaccine development and disease control strategies.

## Data Availability

The datasets presented in this study can be found in online repositories. The names of the repository/repositories and accession number(s) can be found in the article/Supplementary material.

## Supplementary material

SUPPLEMENTARY FIGURE S1 Detection of enteric pathogens in intestinal samples from infected piglets. Representative RT-qPCR and qPCR detection of **(A)** PEDV, **(B)** TGEV, **(C)** 310 PDCoV, **(D)** PoRV, **(E)** GETV, and **(F)** PCV2 were performed in intestinal samples from an affected piglet. PC: positive control.
