# Supplementary material for: Outbreak of porcine deltacoronavirus in pigs in central China, 2024
Source: Front Vet Sci. 2026 Sep 30;13:1934473. doi: 10.3389/fvets.2026.1934473 (PMC13662751; doi:10.3389/fvets.2026.1934473)
Supplement: Supplementary file 1 [file Table_1.DOCX]

Supplementary table 1. PDCoV reference strains used in this study

| Strain | Collection date | Country | Genbank Accession Number |
| --- | --- | --- | --- |
| Michigan/8977/2014 | 2014 | USA | KM012168 |
| USA/Minnesota442/2014 | 2014 | USA | KR265847 |
| USA/NorthCarolina452/2014 | 2014 | USA | KR265858 |
| IN2847 | 2014 | USA | KJ569769 |
| USA/Nebraska209/2014 | 2014 | USA | KR265860 |
| PDCoV/USA/Ohio137/2014 | 2014 | USA | KJ601780 |
| OhioCVM1/2014 | 2014 | USA | KJ769231 |
| KNU16-11 | 2016 | South Korea | KY926512 |
| USA/Minnesota292/2014 | 2014 | USA | KR265864 |
| USA/Illinois273/2014 | 2014 | USA | KR265857 |
| USA/Arkansas61/2015 | 2015 | USA | KR150443 |
| DH1 | 2016 | South Korea | KY354363 |
| GNU-2105 | 2021 | South Korea | OQ566226 |
| PDCoV/Haiti/Human/0256-1/2015 | 2015 | Haiti | MW685623 |
| JSTZ2025 | 2025 | China | PX126649 |
| CH/Jiangsu/2014 | 2014 | China | KY513725 |
| CHN-HB-2014 | 2014 | China | KP757891 |
| CH7328 | 2018 | China | MW816149 |
| CHN-TS1-2019 | 2019 | China | MT663769 |
| CHN-JS-2014 | 2014 | China | KP757892 |
| HNZK-02 | 2018 | China | MH708123 |
| CH/SCQL/2017 | 2017 | China | MW627233 |
| CH/SXD1/2015 | 2015 | China | KT021234 |
| CH/SCBC/2017 | 2017 | China | MW627232 |
| PDCoV/CHGD/2016 | 2016 | China | MH715491 |
| CHN/Tianjin/2016 | 2016 | China | KY065120 |
| PDcov HeN/swine/2015 | 2015 | China | MN942260 |
| CHN/SX-Y/2023 | 2023 | China | PQ373831 |
| CHN-JS-2018 | 2018 | China | OQ551110 |
| CH/Hunan/2014 | 2014 | China | KY513724 |
| HZYH-2019 | 2019 | China | PQ645844 |
| CHN-HG-2017 | 2017 | China | MF095123 |
| CHN/Sichuan/2019 | 2019 | China | MK993519 |
| SCCZ18 | 2018 | China | MT985156 |
| CHN/Sichuan/2017 | 2017 | China | MK211169 |
| SDTA2022 | 2023 | China | PX241255 |
| PDCoV-ZJHZ2024 | 2024 | China | PX492333 |
| GDYF2025-3 | 2025 | China | PX241250 |
| HNXX 2024 | 2024 | China | PX241251 |
| PDCoV/WH/2023 | 2023 | China | OR269935 |
| HuNan-CZ2023 | 2023 | China | PX241253 |
| CH/Sichuan/S27/2012 | 2012 | China | KT266822 |
| CH-01 | 2016 | China | KX443143 |
| P29_15_VN_1215 | 2015 | Thailand | KX998969 |
| PDCoV/Swine/Vietnam/Binh21/2015 | 2015 | Vietnam | KX834352 |
| PDCoV/Swine/Vietnam/HaNoi6/2015 | 2015 | Vietnam | KX834351 |
| P1_13_ST1_0213/PDCoV/0213/Thailand | 2013 | Thailand | KX361343 |
| PDCoV/RBR-1/2016/Thailand | 2016 | Thailand | MZ802777 |
| P1_16_BTL_0115/PDCoV/2016/Lao | 2016 | Laos | KX118627 |
| PDCoV/CBR-1/2016/Thailand | 2016 | Thailand | MZ802772 |
